# Uptake of genetic testing and long-term tumor surveillance in von Hippel-Lindau disease

**DOI:** 10.1186/1471-2350-11-4

**Published:** 2010-01-12

**Authors:** Astrid Rasmussen, Elisa Alonso, Adriana Ochoa, Irene De Biase, Itziar Familiar, Petra Yescas, Ana-Luisa Sosa, Yaneth Rodríguez, Mireya Chávez, Marisol López-López, Sanjay I Bidichandani

**Affiliations:** 1 Department of Biochemistry and Molecular Biology, University of Oklahoma Health Sciences Center, Oklahoma City, OK, USA; 2 Department of Neurogenetics and Molecular Biology, Instituto Nacional de Neurología y Neurocirugía Manuel Velasco Suárez, Mexico City, D.F., Mexico; 3 Department of Mental Health, Johns Hopkins Bloomberg School of Public Health, Johns Hopkins University, Baltimore, MD, USA; 4 Division of Psychiatry, Instituto Nacional de Neurología y Neurocirugía Manuel Velasco Suárez, Mexico City, D.F., Mexico; 5 Department of Neuropsychology and Support Groups, Instituto Nacional de Neurología y Neurocirugía Manuel Velasco Suárez, Mexico City, D.F., Mexico; 6 Department of Biological Systems, Division of Biological and Health Sciences, Universidad Autónoma Metropolitana-Xochimilco, Mexico City, Mexico

## Abstract

**Background:**

von Hippel-Lindau (VHL) disease is a hereditary cancer syndrome caused by germline mutations in the *VHL *gene. Patients have significant morbidity and mortality secondary to vascular tumors. Disease management is centered on tumor surveillance that allows early detection and treatment. Presymptomatic genetic testing is therefore recommended, including in at-risk children.

**Methods:**

We tested 17 families (n = 109 individuals) for *VHL *mutations including 43 children under the age of 18. Personalized genetic counseling was provided pre and post-test and the individuals undergoing presymptomatic testing filled out questionnaires gathering socio-demographic, psychological and psychiatric data. Mutation analysis was performed by direct sequencing of the *VHL *gene. Mutation-carriers were screened for VHL disease-related tumors and were offered follow-up annual examinations.

**Results:**

Mutations were identified in 36 patients, 17 of whom were asymptomatic. In the initial screening, we identified at least one tumor in five of 17 previously asymptomatic individuals. At the end of five years, only 38.9% of the mutation-carriers continued participating in our tumor surveillance program. During this time, 14 mutation carriers developed a total of 32 new tumors, three of whom died of complications. Gender, education, income, marital status and religiosity were not found to be associated with adherence to the surveillance protocol. Follow-up adherence was also independent of pre-test depression, severity of disease, or number of affected family members. The only statistically significant predictor of adherence was being symptomatic at the time of testing (OR = 5; 95% CI 1.2 - 20.3; p = 0.02). Pre-test anxiety was more commonly observed in patients that discontinued follow-up (64.7% vs. 35.3%; p = 0.01).

**Conclusions:**

The high initial uptake rate of genetic testing for VHL disease, including in minors, allowed the discontinuation of unnecessary screening procedures in non mutation-carriers. However, mutation-carriers showed poor adherence to long-term tumor surveillance. Therefore, many of them did not obtain the full benefit of early detection and treatment, which is central to the reduction of morbidity and mortality in VHL disease. Studies designed to improve adherence to vigilance protocols will be necessary to improve treatment and quality of life in patients with hereditary cancer syndromes.

## Background

von Hippel-Lindau (VHL) disease is an autosomal dominant disease characterized by predisposition to multiple tumors, which include cerebellar and spinal hemangioblastomas, retinal angiomas, benign renal and pancreatic cysts, renal cell carcinoma and pheochromocytoma. The responsible gene is *VHL*, a tumor suppressor located on chromosome 3p25-26, which encodes an ubiquitin ligase that is involved in the cellular response to hypoxia [1,2].

VHL disease affects a wide variety of organs throughout life, and it is associated with high morbidity and mortality. The prognosis has improved in recent years, partly because of better surgical techniques, but primarily because management of VHL disease families is now centered on early detection and treatment of tumors through periodic surveillance programs. Presymptomatic genetic testing has been central to this goal. Once a causative mutation is identified in an affected family member, all at-risk individuals are tested and mutation carriers undergo periodic tumor screening while non-carriers avoid any unnecessary screening procedures [3].

Presymptomatic genetic testing is a complex process because of the potential psychological, social and economic implications of receiving an abnormal result, and the difficulties associated with adapting to normal results (survivor guilt) [4]. This is even more complicated in the case of minors [5,6]. However, there is general agreement that in the case of diseases in which the potential benefits of early detection significantly outweigh the harms associated with the test, it is reasonable to offer presymptomatic genetic testing to children [7-9]. Examples of such diseases include multiple endocrine neoplasia (MEN) and familial adenomatous polyposis (FAP). In MEN, genetic testing is recommended before the age of 5, given the risk of early medullary thyroid carcinoma and the availability of prophylactic thyroidectomy, [3,10] while in FAP, the recommended age of genetic testing is between ages 10 and 12, when colonoscopic or sigmoidoscopic surveillance is initiated [11]. In both these diseases, if the mutation in the family is known and the child is determined to be a non-carrier, reassurance can be given that it is safe to discontinue tumor surveillance and prophylactic surgery is thus avoided [3,10-12].

In the case of VHL disease, the tumor surveillance program includes annual screening for central nervous system hemangioblastomas, measurement of catecholamines and imaging of the abdomen, as well as semiannual screening for retinal angiomas [13,14]. While the average age of onset of VHL disease is in the third decade of life, some patients develop their first tumors before their tenth birthday, and occasionally even in infancy. Therefore, presymptomatic genetic testing is justified in minors at-risk for VHL disease so that tumor surveillance may be initiated early in mutation-carriers, while non-carriers may avoid unnecessary screening procedures [12,15].

We describe the uptake of diagnostic and presymptomatic genetic testing in a series of 109 individuals for VHL disease, the results of the initial screening for VHL-related tumors in mutation-carriers, and the uptake and subsequent adherence to tumor surveillance over a five year period. An attempt was made to identify the factors influencing their adherence to a long-term follow-up program for hereditary cancer.

## Methods

**Figure 1:**
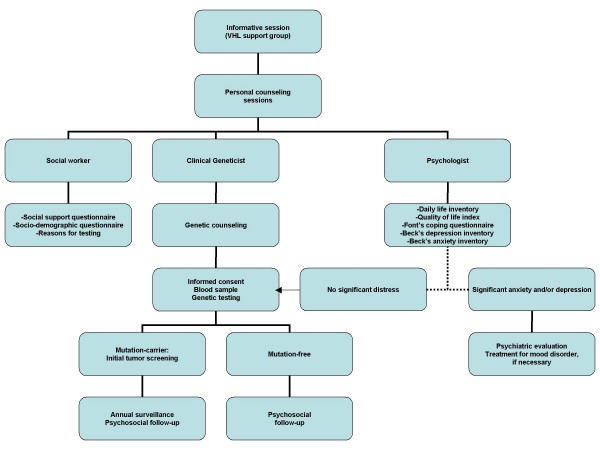
**Scheme for genetic counseling and testing in our cohort of families with VHL disease**.

Seventeen families participated in the present study. The proband in each family was ascertained at the National Institute of Neurology and Neurosurgery (INNN) in Mexico City in 2002, with a diagnosis of VHL disease or possible VHL disease. Some of these families were included in a previous report [16]. The diagnosis of definite VHL disease was established when the patients fulfilled the modified clinical criteria according to Neumann et al. [17]. Possible VHL disease was considered when the patient had a VHL disease-related tumor, but the family history was either unclear or impossible to corroborate. The overall scheme for genetic testing is shown in Figure 1.

Genetic counseling was provided to each proband after obtaining informed consent according to a protocol approved by the IRB at INNN. We extracted DNA from peripheral blood samples and analyzed the coding-sequence, promoter region and all exon-intron boundaries of the *VHL *gene for mutations, as previously described [16]. This strategy does not detect large deletions, however in our series of patients we have successfully identified mutations in 85-90% definite VHL families.

When the result of the genetic test was revealed to the proband, implications for the rest of the family were also discussed, and genetic counseling was offered to all relatives at risk. The initial counseling session was held as part of a pilot VHL disease support group at INNN, and it included details about VHL disease and its natural history, inheritance, and information about genetic testing and tumor surveillance. A summary of the information was provided in writing at the end of the session and all interested individuals underwent further personal counseling sessions with a certified medical geneticist.

Genetic testing of the family members was conducted following the recommendations of the American Society of Clinical Oncology for genetic testing for cancer susceptibility, [18] and according to an IRB approved protocol at INNN. All adults signed an informed consent form for themselves and for their minor children; assent of the child was also obtained if they were between 11 and 17 years old [7]. Pre-test counseling included interviews with the geneticist, social worker, and a clinical psychologist experienced in presymptomatic genetic testing. Adults completed a series of questionnaires: personal history and socio-demographic data, reasons for taking the genetic test, Daily Life Inventory, Life Quality Index, social support questionnaire, Font's coping questionnaire, Beck's Depression Inventory and Beck's Anxiety Inventory.

If significant distress was noted at the interview or in the questionnaires, a further evaluation was performed by an experienced psychiatrist and, if necessary, the test was postponed and/or treatment for the condition was initiated.

All mutation carriers underwent an initial screening for VHL disease-related tumors. Appointment for the following year's screening was set up upon completion of the initial screen by the social worker, and this procedure was repeated annually according to our tumor surveillance program, which was adapted form the recommended guidelines of the VHL family alliance, [13,14,19] and is shown in Table 1.

**Table 1:** Tumor surveillance program in *VHL *gene mutation-carriers

Age (y)	Physical exam	Fundoscopic examination	Urinary catecholamines	Brain/spinal imaging	Abdominal imaging
0 - 2	12 mo	24 mo	------	------	-----
2-10	12 mo	12 mo	12 mo	12 mo	-----
11-19	12 mo	6 mo	12 mo	12 mo	12 mo (US)
>20	12 mo	6 mo	12 mo	12 mo	12 mo (MRI)

Mo: months; US: Ultrasonographic screening; MRI: Magnetic resonance imaging

Adapted from: VHL family alliance http://www.vhl.org/handbook/vhlhb4.php#Suggested,

Choyke et al, 1995 and Lonser et al, 2003.

Crude and adjusted odds ratios (ORs) were estimated by logistic regression models. Tests for adherence to surveillance protocol variables and statistical significance were evaluated using two-sided design-based tests at the 0.05 level of significance. Analysis was done using STATA Intercooled version 10.1 (StataCorp. *Stata Statistical Software: Release 10*. 2007;10.1)

## Results

### Uptake of presymptomatic testing

Our genetic testing program for VHL disease included 17 families, ten of which had definite VHL disease and seven had possible VHL disease. Testing of the proband in each family allowed us to identify mutations in the *VHL *gene in 9/10 definite VHL disease families and 3/7 possible VHL disease families. It is possible that the family diagnosed with definite VHL disease in which we did not identify a mutation has a large gene deletion, which would have been missed by our current testing strategy[16].

**Figure 2:**
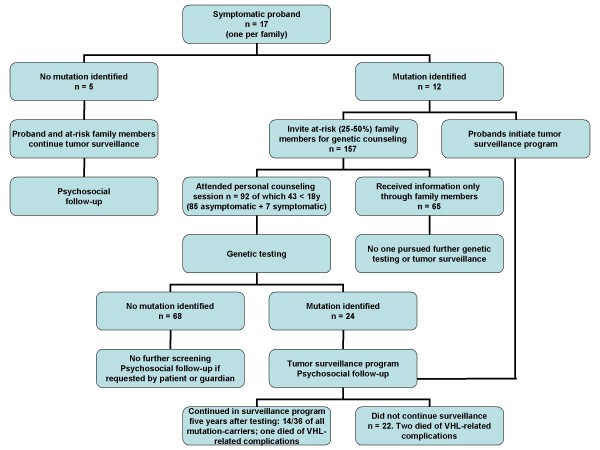
Uptake of predictive testing in our cohort of 17 families with VHL disease.

A detailed overview of the uptake of presymptomatic testing in our cohort is outlined in Figure 2. Analysis of the pedigrees of the 12 families with known mutations revealed that there were 157 family members at 25 - 50% risk; 92 of them underwent genetic testing. In 85 cases, the testing was presymptomatic because the individuals were asymptomatic; the remaining seven already had some manifestation of VHL disease and received confirmatory diagnostic testing. Almost half of the at-risk family members tested were children under the age of 18 (43/92, 47%).

Uptake of the test was high and we did not experience any drop-out prior to the delivery of the genetic test results. Indeed, all of the 92 family members that attended personal counseling sessions consented to genetic testing. However, at-risk family members that only received information through their affected relatives, and who did not come to the information and counseling sessions, did not express any further interest in genetic testing or periodic clinical surveillance (n = 65; 41%).

The genetic testing results revealed a total of 36 mutation carriers, with a median age of 20.5 years (range 1 - 45 years; SD ± 11.41 years). They were 12 probands and 24 at-risk family members, 17 of whom were asymptomatic at the time of testing (Table 2).

**Table 2:** Results of genetic testing and tumor surveillance in *VHL *gene mutation-carriers

Family	Mutation	Age	Follow-up	Status pre-test	Status 1st. Screening	Final status§
1	p.F76del	>18	Y	Affected	3 Hb, KC	+ PC
		>18	Y	Affected	3 Hb	Same
		>18	Y	Affected	4 Hb	Same
		<18	Y	Asymptomatic	Asymptomatic	Asymptomatic
6	c.99_100InsA*	>18	Y	Affected	1 Hb, Bilat RA	Same
		<18	N	Affected	4 Hb, Bilat RA, KC, PC	+ 1 Hb, ✞
		>18	N	Affected	1 Hb, Bilat RA, KC, PC	+ 2 RCC
		>18	N	Asymptomatic	1 Hb	+ 1 Hb
		<18	N	Asymptomatic	Asymptomatic	Asymptomatic
		<18	N	Asymptomatic	Asymptomatic	Asymptomatic
7	c.99_100InsA*	>18	N	Asymptomatic	EC	Same
		<18	N	Asymptomatic	Asymptomatic	Asymptomatic
		>18	N	Affected	1 Hb, 1 RCC, KC, PC	+ 1 RCC
		<18	N	Asymptomatic	Asymptomatic	+ 1 Hb
		>18	N	Asymptomatic	3 Hb, KC, PC	+ 1 RCC
		<18	N	Asymptomatic	Asymptomatic	Asymptomatic
		>18	N	Asymptomatic	1 Hb, KC, PC	Same
		>18	N	Affected	1 Hb	+ 1 Hb
		<18	Y	Affected	1 Hb	Same
		<18	N	Asymptomatic	Asymptomatic	Asymptomatic
		>18	N	Asymptomatic	1 Hb	+ KC, PC, 1 RCC
		>18	N	Affected	1 Hb, KC, PC	Same
		<18	N	Asymptomatic	Asymptomatic	Asymptomatic
12	p.D121G	>18	Y	Affected	2 Hb, Bilat RA, KC, PC, 1 RCC, 1 pheo	Same
16	p.R161X	>18	N	Affected	3 Hb, KC, PC	Same
19	p.P86S	>18	N	Affected	1 Hb, KC, PC, 1 RCC	+ 1RCC, 1 Hb, ELST, ✞
20	p.C162F	>18	Y	Affected	2 Hb, Unilat RA, KC, PC, 1 pheo	+ 1 RCC, 1 pheo
21	p.R82P	>18	Y	Affected	1 Hb	+ 2 Hb, Bilat RA, KC, PC, 1 RCC, ✞
22	c.56_57DupInvCGGGAGGC*	>18	N	Affected	1 Hb	Same
		>18	N	Asymptomatic	Asymptomatic	Asymptomatic
		>18	N	Asymptomatic	Asymptomatic	Asymptomatic
23	p.E134X*	>18	Y	Affected	2 Hb, Bilat RA, KC, 1 RCC	Same
		>18	Y	Asymptomatic	Asymptomatic	Asymptomatic
		>18	Y	Asymptomatic	Asymptomatic	Asymptomatic
43	p.P86S	>18	Y	Affected	1 Hb	+ 3 Hb, 2 RCC, PCA, 1 Pheo
53	p.L89P	>18	Y	Affected	KC, PC	+ 1 Hb

* Novel mutation. Y = Yes, N = No; Hb = Hemangioblastoma, KC = Kidney cysts, PC = Pancreatic cysts, EC = Epididymal cysts, RCC = Renal cell carcinoma, Pheo = Pheochromocytoma, ELST = Endolymphatic sac tumor, PCA = Pancreatic cancer. ✞ Deceased. §Final status was determined by the results of the most recent surveillance testing or, if the patient had discontinued surveillance final status was determined by phone re-contact or if the patient returned seeking medical attention for new symptoms.

Genotype-phenotype analysis confirmed that families carrying protein truncating mutations had a VHL disease type 1 phenotype, and families with missense mutations had VHL disease with pheochromocytoma (VHL type 2). The exception was family 19 (P86S missense mutation) in which the only affected individual died without evidence of pheochromocytoma.

### Tumor surveillance and follow-up

After disclosure of the test results, the mutation carriers were offered screening for VHL disease related tumors and follow-up with annual examinations. All of them (n = 36) undertook the initial screening, in which we identified one or more VHL disease tumors in 5/17 previously asymptomatic mutation carriers (Table 2).

Five years after the 36 mutation-carriers underwent the initial tumor screening, only 14 (38.9%; from eight families) continued with annual surveillance. Since the time of genetic testing, 14 mutation-carriers have developed a total of 32 new VHL disease-related tumors (Table 2). One child died of complications of an inoperable bulbar hemangioblastoma and two adults died of complications of metastatic renal cancer. Individuals in whom more than one tumor was identified during the follow-up period were almost twice as likely to die compared to those who did not develop further tumors (OR = 1.8; 95% CI = 1.1 - 3.1; p = 0.03).

In order to identify factors that may have influenced the uptake and adherence to the tumor surveillance program, we analyzed the clinical (Table 3), psychological and socio-demographic features (Table 4) of the mutation carriers. Whereas clinical information was available for all 36 patients, the complete set of socio-demographic and psychological tests was available for 17 adults and may represent a limitation of the study.

**Table 3:** Clinical correlates of adherence to tumor surveillance program

Clinical features	n	Follow-up (+)	Follow-up (-)	p
Gender	36			n.s.
Male	13	5	8	
Female	23	9	14	
Pre-test clinical status	36			**p = 0.02**
Asymptomatic		3	14	
Symptomatic		11	8	
Pre-test depression^†^	17			n.s.
Yes		1	3	
No		6	7	
Pre-test anxiety^‡^	17			**p = 0.01**
Yes		0	7	
No		6	4	
Offspring	36			n.s.
One or more children		7	9	
No children		7	13	
Clinical status of offspring^†‡^	16			n.s.
Affected		2	3	
Unaffected		5	6	
Mutation status of offspring^†‡^	16			n.s.
Mutation-carrier		5	6	
Non mutation-carrier		2	3	

Follow-up (+): Patients that continued in the tumor surveillance program five years after genetic testing. Follow-up (-): Patients that had abandoned the tumor surveillance program five years after genetic testing.

^† ^Score ≥ 10 on Beck's Depression Inventory. Includes individuals that completed the psychological questionnaires (n = 17)

^‡ ^Score ≥ 8 on Beck's Anxiety Inventory. Includes individuals that completed the psychological questionnaires (n = 17)

^†‡ ^Includes mutation-carriers who had children (n = 16)

**Table 4:** Socio-demographic correlates of adherence to tumor surveillance program

Sociodemographic feature	n	Follow-up (+)	Follow-up (-)	p
Religious				n.s.
No	10	4	6	
Yes	7	3	4	
Education				n.s.
< National average	7	3	4	
≥ National average	10	4	6	
Marital Status				n.s.
Married^†^	10	4	6	
Not married^‡^	7	3	4	
Income				n.s.
< National poverty line	13	5	8	
≥ National poverty line	4	2	2	

^† ^Includes married, civil unions and cohabitation

^‡ ^Includes single, divorced and widowed

Individuals who were symptomatic before the molecular test were 5 times more likely to continue the surveillance program (OR = 5; 95% CI 1.2 - 20.3; p = 0.02), which was maintained even after adjustment for the clustering of observations (OR = 5.0, CI 95%= 1.37-18.29; p = 0.02). On the other hand, significant pre-test anxiety was more common amongst the individuals that prematurely dropped out of surveillance (64.7% vs. 35.3%; p = 0.01). Follow-up was not found to be associated with having or not having children, the mutation status or affectedness of the children, and there was also no relationship with pre-test depression (Table 3). Gender, education, income, marital status, and religiosity, did not modify the likelihood of continuing the tumor surveillance beyond the initial screening (Table 4).

In the single mutation-free family with definite VHL disease based on clinical criteria we suggested that the proband and family members at 50% risk continue with the annual tumor screening. We also suggested long-term surveillance for the probands of the possible VHL disease families.

## Discussion

We describe the five-year follow-up of a series of 109 individuals that underwent presymptomatic genetic testing for VHL disease. We identified a total of 36 mutation carriers, of which 17 were asymptomatic at the time of testing. Initial screening for tumors revealed that five presumptive asymptomatic individuals already had one or more VHL disease-related tumor(s) and a total of 32 new tumors were identified during the five-year follow-up.

There are few reports of long-term follow-up of individuals that have been tested for hereditary cancer syndromes, and very little is known about the uptake and adherence to follow-up in VHL disease. Further analyses of long-term outcomes are necessary, given the generalized assumption amongst specialists providing genetic testing that communication of cancer risk information leads to a subsequent modification in behavior [20]. Recently, Beery et al made a systematic review of published studies describing the health-promotion and risk-reduction behavioral outcomes following presymptomatic genetic testing in adult-onset disorders [21]. However, their analysis included short-term follow-up for only two hereditary cancer syndromes: hereditary breast and ovarian cancer syndrome (HBOC) and hereditary non-polyposis colon cancer (HNPCC) [21,22]. One report of families with VHL disease and other inherited cancer predisposing syndromes showed that the uptake of presymptomatic testing for VHL disease was 70% of the at-risk family members; however, the adherence of mutation-carriers to long term surveillance program was not reported [23].

In our series of VHL disease families, initial interest and uptake of presymptomatic genetic testing for VHL disease was very high, but less than half of the mutation carriers engaged in risk-reduction behaviors through the five year follow-up period. Whenever a patient was diagnosed with VHL disease, genetic counseling was provided to explain the risks for the rest of the family members. Once a VHL disease-causing mutation was identified, we contacted the patient again and stressed the importance of sharing this information with their relatives. We organized informative sessions for extended VHL disease families, and personal genetic counseling for all relatives at risk. All of the at-risk relatives that attended these sessions chose to be tested for *VHL *mutations and to have their children tested as well. This is different from what was observed in patients at the same hospital who were offered presymptomatic genetic testing for Huntington disease. In a series of 373 families with Huntington disease followed over 14 years at the INNN, only 11.5% had one or more individuals that underwent presymptomatic testing, in contrast with 58% of the at-risk VHL disease family members (p < 0.001). This difference in uptake of presymptomatic genetic testing is most likely attributable to the lack of preventive and therapeutic options available in Huntington disease [24,25].

A relevant factor in determining uptake of genetic testing was whether they had personal genetic counseling. When at-risk family members were only informed about the possibility of testing through a relative and/or written material, they were unlikely to pursue further counseling or testing. This differs from what has been reported in other populations, where at-risk family members contacted through telephone interviews or letters had a positive attitude and high uptake rate of genetic testing [23,26,27].

The poor adherence to long-term surveillance also contrasts with the observations of another series of Mexican patients with hereditary cancer syndromes (HBOC or HNPCC) enrolled in a similar surveillance program at the Instituto Nacional de Cancerologia in Mexico City, Mexico. In that cohort, >95% of patients with HBOC or HNPCC have continued with long-term follow-up (Dr. Silvia Vidal, *personal communication*), ruling out population-specific causes for the drop-out in VHL disease surveillance.

Forty-three of the tested individuals were children under the age of 18. In general terms, it has been suggested that presymptomatic genetic testing should not be undertaken for children unless the benefits outweigh potential harm [7,9]. In the case of VHL disease, as well as multiple endocrine neoplasia syndromes and familial adenomatous polyposis, it is considered justifiable to perform presymptomatic testing of minors based on the early age of onset of some of the tumors, the potential of reducing morbidity by early treatment, and to prevent non-carriers from undergoing expensive and invasive annual screening [8,11,12]. In our series, of the children that were mutation carriers (n = 10), three had tumors and one died of complications of a recurrent bulbar hemangioblastoma. On the other hand, 33 at-risk children were found to not carry the mutation and were therefore relieved from the burden of life-long screening for VHL disease-related tumors.

We encouraged continued surveillance for families in whom we were unable to identify *VHL *mutations. This recommendation was based on literature reports that have calculated that patients with an apparently isolated VHL-related tumor and no detectable VHL mutation have a risk of ≈ 5% of developing subsequent VHL-related tumors within 10 years of the diagnosis of their first tumor [28]. Interestingly, the adherence to follow-up surveillance was lower in the families with confirmed *VHL *mutations (38.9%) than in families with definite or possible VHL disease in whom no mutation was identified (63.5%; p = 0.01). They include the proband of the definite VHL disease family and her three daughters, one of which has since developed a cerebellar hemangioblastoma. Although the reason for this difference is unknown, one may speculate that the uncertainty of knowing their carrier-status may have prompted them to stay in the surveillance program; it would be interesting to design a future study to address this issue.

Most socio-demographic and psychiatric variables analyzed did not correlate with follow-up adherence. However, individuals who were already symptomatic at the time of testing had a higher likelihood of adhering to long-term follow-up (OR = 5; 95% CI 1.2 - 20.3; p = 0.02), and those who had significant pre-test anxiety tended to abandon the follow-up program (p = 0.01). Interestingly, in the case of families with several mutation-carriers, most of the family members tended to take the same stance towards long-term surveillance. Families 1 and 23 had all their mutation-carriers continue with the follow-up program five years after testing, while Families 6, 7 and 22 did not continue (with the exception of one adult in Family 6 and one child in Family 7). This is in contrast with a previous study of VHL disease in which attitudes towards genetic testing for VHL disease in the patient and offspring were personal decisions and not those of a group [29].

Another factor that seemingly promoted active participation in follow-up testing was if the family leader, i.e., the person encouraging family members to attend, and often also the person that coordinated appointments at the hospital, was a female. Unfortunately, the small number of families with male leaders did not allow statistical confirmation of this trend. One family (Family 7) further helped to support this notion: at the beginning of the program, the wife of a proband actively promoted education and participation of all family members in the VHL disease program. However, after her death the family was lost to follow-up and several attempts to contact them were not effective in bringing them back into the program. These findings are consistent with previous analyses of psycho-social factors influencing genetic testing, which have suggested that families led by a matriarch have higher levels of participation and follow-up than families led by a patriarch. It has also been suggested that engaging a matriarch in the process of risk communication and management may improve overall uptake [30,31].

Finally, we considered the economic burden of the surveillance program, which includes multiple medical procedures, some of them potentially costly, as an impediment for continued surveillance. However, subjects enrolled in our VHL disease screening program at INNN were specifically exempt from ≥ 90% of the costs of all the screening tests, thus eliminating financial constraints as a cause for leaving the program. Indeed, we also scheduled and synchronized all tests to be performed during a single hospital visit, and offered convenient re-scheduling, when needed. Another potential explanation for the lack of adherence to long-term follow-up is that patients report being overwhelmed by the disease which, in contrast to other hereditary cancer syndromes, has an early onset and affects a wide variety of organs. However, our analysis did not reveal a difference in adherence to surveillance in patients with more severe disease (measured as number of tumors) and continuation in the surveillance program. This is a little explored aspect of genetic testing for hereditary cancer, and warrants further analysis.

## Conclusions

The high initial uptake rate of genetic testing for VHL disease, including in minors, allowed the discontinuation of unnecessary screening procedures in non mutation-carriers. However, mutation-carriers showed poor adherence to long-term tumor surveillance. Therefore, many of them did not obtain the full benefit of early detection and treatment, which is central to the reduction of morbidity and mortality in VHL disease. The factors that influence the uptake of risk-reducing and health-promoting behaviors need to be analyzed in larger, prospective follow-up series, in order to determine how to realize the potential benefits of presymptomatic genetic testing and systematic tumor surveillance.

## Abbreviations used

VHL: von Hippel-Lindau disease; MEN: Multiple endocrine neoplasia; FAP: Familial adenomatous polyposis; HBOC: Hereditary breast and ovarian cancer; HNPCC: Hereditary non-polyposis colon cancer; INNN: National Institute of Neurology and Neurosurgery, Mexico City, Mexico; IRB: Internal Review Board.

## Competing interests

The authors declare that they have no competing interests.

## Authors' contributions

AR: Conceived the study, participated in its design and coordination, recruited patients, genetic counseling, clinical follow-up and drafted the manuscript. EA: Participated in the design and coordination of the study, in recruiting patients, genetic counseling and clinical follow-up. AO: Participated in the design of the study and coordinated all patient related activities as well as the tumor surveillance protocol; performed the socio-demographic evaluation of patients. IDB: Carried out the molecular genetic studies. PY: Participated in the molecular genetic studies. IF: Statistical analysis. ALS: Psychiatric assessment, counseling and treatment of patients. YR: Psychological evaluation and counseling of patients. MC: Psychological evaluation and counseling of patients. MLL: Design of molecular genetic testing strategy, helped draft the manuscript. SIB: Analysis and interpretation of data, critical revision and modification of the manuscript. All authors read and approved the final manuscript.

## Pre-publication history

The pre-publication history for this paper can be accessed here:

http://www.biomedcentral.com/1471-2350/11/4/prepub
